# Montage-guided fusion network for long-term scalp electroencephalography seizure detection

**DOI:** 10.3389/fninf.2026.1942480

**Published:** 2026-09-01

**Authors:** Hongtao Yin, Xin Yin

**Affiliations:** 1 Department of Neurology, Zibo Central Hospital, Zibo, Shandong, China; 2 First Clinical Medical College, Shandong Medical and Pharmaceutical University, Yantai, Shandong, China

**Keywords:** bipolar montage, deep learning, EEG signal processing, epileptic seizure detection, graph transformer, scalp EEG

## Abstract

**Introduction:**

Automatic seizure detection from long-term scalp electroencephalography (EEG) can reduce the burden of manual EEG review. However, multi-derivation EEG is commonly processed as a collection of input channels without explicitly modeling the structured relations defined by the bipolar montage.

**Methods:**

In this study, we propose MGF-Net, a montage-guided fusion network for seizure detection from long-term scalp EEG. MGF-Net represents 18 fixed bipolar derivations as nodes in a relation-labeled graph. The graph encodes same-chain adjacency, left-right correspondence, and shared-electrode relations derived from the bipolar montage. After epoch-wise robust normalization and wavelet-based signal reconstruction are applied independently to each epoch, a shared one-dimensional convolutional neural network extracts temporal features from each derivation. A relation-aware graph Transformer then performs feature fusion across derivations using relation-specific key and value projections. Attention-based graph pooling generates an epoch representation for ictal probability estimation, and post-processing converts epoch-wise probabilities into seizure-event detections.

**Results:**

Experiments on the CHB-MIT dataset show that MGF-Net achieves a segment-level accuracy of 98.38%, sensitivity of 93.18%, and specificity of 98.41%. At the event level, the proposed method detected all 65 annotated test events, achieving a sensitivity of 100.00%, a false detection rate of 0.82/h, and a latency of 2.03 s.

**Discussion:**

These results demonstrate MGF-Net's effectiveness for seizure detection from long-term scalp EEG.

## 1 Introduction

Epilepsy is a common neurological disorder characterized by recurrent seizures associated with abnormal neuronal activity (Asadi-Pooya et al., 2023). Long-term scalp electroencephalography (EEG) provides a non-invasive recording of brain electrical activity and remains important for epilepsy diagnosis and seizure monitoring (Li et al., 2024). However, reviewing long-term EEG recordings is labor-intensive and requires substantial clinical expertise, especially when seizure events are sparse relative to background activity and when seizure duration and morphology vary across patients and recordings (Wong et al., 2025). Automatic seizure detection systems can assist clinicians by identifying candidate seizure events and reducing the burden of manual EEG review.

Conventional EEG-based seizure detection pipelines commonly rely on engineered features extracted from the time, frequency, or time–frequency domains, followed by machine-learning classifiers (Handa et al., 2024; Slama et al., 2025). In contrast, recent deep learning methods enable end-to-end representation learning from EEG recordings. Convolutional neural networks (CNNs) can extract local waveform patterns, whereas recurrent networks and temporal convolutional networks can capture temporal dependencies across samples or feature sequences. Transformer-based models can further model long-range dependencies, while graph-based approaches provide an explicit mechanism for representing inter-derivation structure (Dong et al., 2024; Li C. et al., 2025). Although these approaches can integrate information from multiple derivations, cross-derivation interactions in many existing methods are modeled through joint multichannel representations, generic attention mechanisms, predefined adjacency structures, or single-relation graph connectivity, while construction-specific relationships between bipolar derivations are not commonly represented as explicit variables. Consequently, the model may not distinguish whether two derivations are adjacent within the same montage chain, correspond across the left and right hemispheres, or share an electrode.

**Figure 1:**
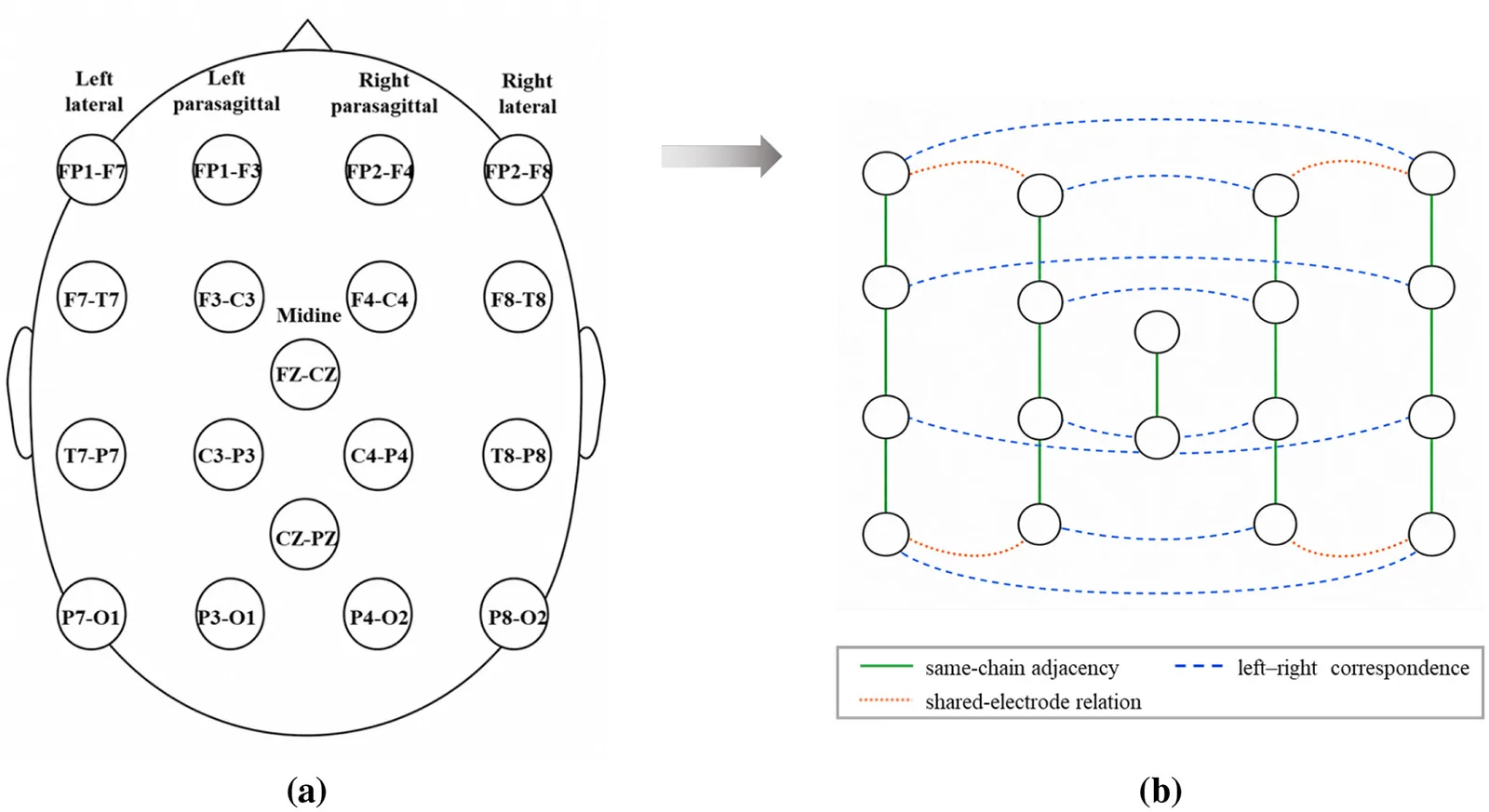
Montage-derived relation prior used in MGF-Net. **(a)** Layout of the 18 bipolar derivations retained from the fixed bipolar montage. **(b)** Corresponding relation-labeled montage graph, in which each bipolar derivation is represented as a node. Green solid, blue dashed, and orange dotted edges denote same-chain adjacency, left–right correspondence, and shared-electrode relations, respectively.

Scalp EEG recordings are commonly inspected using bipolar montages, in which each derivation represents the voltage difference between two electrodes (Xu et al., 2025). Beyond specifying the input derivations, the arrangement of a bipolar montage provides a fixed relational structure among derivations (Wang et al., 2026). Adjacent derivations within the same chain correspond to consecutive segments of a bipolar sequence. Derivations at corresponding positions in the left and right chains encode left–right correspondence, whereas derivations sharing one electrode define local cross-chain relations. These montage-derived relations are illustrated in Figure 1. These relations do not estimate anatomical or functional connectivity. Instead, they constitute a fixed montage-derived structural prior that guides how the model compares and integrates features across derivations.

Motivated by this observation, we propose MGF-Net, a montage-guided fusion network for seizure detection from long-term scalp EEG. MGF-Net applies a shared one-dimensional CNN to each bipolar derivation to extract temporal features in a common representation space. The resulting features are represented as nodes in a graph constructed from a fixed set of 18 bipolar derivations. A relation-aware graph Transformer then conditions cross-derivation information exchange on the montage-defined relation labels, so that interactions depend not only on the current derivation features but also on how the corresponding bipolar derivations are related within the montage. Attention-based graph pooling combines the updated derivation features into a single feature vector for each epoch, which is used to estimate the ictal probability. At the recording level, post-processing converts epoch-wise probabilities into seizure-event detections.

The main contributions of this study are as follows:

- We formulate multi-derivation scalp EEG seizure detection as a relation-labeled graph learning problem. The proposed montage graph represents 18 fixed bipolar derivations as nodes and distinguishes same-chain adjacency, left–right correspondence, and shared-electrode relations, thereby providing an explicit montage-based structural prior.
- We propose MGF-Net, which combines a shared 1D CNN with a relation-aware graph Transformer. The shared CNN extracts temporal features from individual derivations in a common representation space, while relation-specific key and value projections enable montage-guided information exchange across derivations.
- We develop a recording-level seizure detection pipeline that combines graph pooling with post-processing to convert epoch-wise probabilities into seizure-event detections. The proposed method is evaluated on long-term scalp EEG recordings using both segment-level and event-level criteria.

## 2 Related work

Automatic seizure detection from long-term EEG has evolved from feature-engineering pipelines toward deep learning frameworks that learn representations directly from multichannel recordings. Earlier methods commonly relied on time–frequency analysis and manually designed descriptors before classification. Zhang et al. (2022) applied Db4 wavelet decomposition to multichannel EEG, extracted relative-energy features from selected wavelet components, and used a bidirectional gated recurrent unit (Bi-GRU) network to model temporal dependencies across short time slices within each 4-s epoch. Zhou et al. (2023) developed a real-time seizure detector based on STFT-derived EEG representations and a CNN–LSTM architecture, followed by output smoothing for event generation. These approaches can capture spectral characteristics associated with seizure activity, but their performance depends partly on the selected signal representation and the subsequent strategy for integrating information across channels (Zhong et al., 2023).

Recent studies have increasingly combined convolutional feature extraction with recurrent or attention-based sequence modeling (Li Q. et al., 2025). Li et al. (2020) proposed a fully convolutional nested LSTM framework, in which convolutional blocks learn hierarchical features from EEG segments and nested LSTM units model temporal dependencies. Dong et al. (2024) introduced a TCN-BiLSTM model that processes filtered multichannel EEG using temporal convolutional blocks with multiple dilation factors and then applies bidirectional LSTM layers for sequence modeling. (Li C. et al. 2025) proposed CNN-Informer, which combines DWT-based filtering, convolutional feature extraction, and Informer encoders with ProbSparse attention to model long-range dependencies efficiently. Zhu and Wang (2023) similarly used an EEGNet-based convolutional front end to extract temporal and spatial features from multichannel EEG, followed by a Transformer encoder for sequence-level dependency modeling. Collectively, these methods demonstrate the benefit of integrating local waveform feature extraction with temporal context modeling. When these models integrate information across channels, the interactions are generally learned through joint convolutional representations, channel-wise feature maps, or generic attention mechanisms rather than through explicitly typed relations derived from the construction of bipolar derivations.

Graph-based methods provide a more explicit mechanism for incorporating inter-channel structure into seizure detection (Lian and Xu, 2023). Covert et al. (2019) treated scalp EEG as a structural time series and proposed temporal graph convolutional networks (TGCNs), in which feature-extraction operations are localized and shared across both the temporal dimension and graph neighborhoods. Their framework represents lead-level topology using a predefined binary adjacency matrix. Zhao et al. (2021) constructed a graph from the Pearson correlation matrix of raw EEG channels and employed a linear graph convolutional network with focal loss for seizure detection. These studies demonstrate that graph representations can exploit cross-channel dependence beyond independent channel processing. However, their graph constructions encode either lead-level topology or correlation-derived channel connectivity, rather than explicitly distinguishing multiple construction-specific relation types between bipolar derivations, such as same-chain adjacency, left–right correspondence, and shared-electrode relations.

An alternative multichannel fusion strategy is montage-consistent channel serialization. Wang et al. (2026) proposed MCLF, which applies a shared CNN to individual bipolar derivations, arranges the resulting embeddings in a montage-consistent order, and integrates them through liquid-state evolution to produce an epoch-level representation. This design preserves a fixed channel order during fusion and enables state-based accumulation of multichannel evidence. However, channel serialization does not explicitly specify whether a pair of derivations is adjacent within the same bipolar chain, corresponds across hemispheres, or shares an electrode.

Based on the above literature, MGF-Net represents bipolar derivations as nodes in a relation-labeled montage graph. Instead of representing cross-derivation structure solely through a generic adjacency pattern, correlation-derived connectivity, planar channel layout, or an ordered channel sequence, MGF-Net explicitly distinguishes same-chain adjacency, left–right correspondence, and shared-electrode relations. A relation-aware graph Transformer then combines derivation-specific temporal representations while conditioning cross-derivation information exchange on these montage-derived relation labels. Thus, unlike graph formulations in which edges primarily indicate whether or how strongly two EEG channels are connected, MGF-Net additionally represents the type of montage-defined relationship between bipolar derivations and incorporates this relation type directly into feature fusion.

## 3 Materials and methods

**Figure 2:**
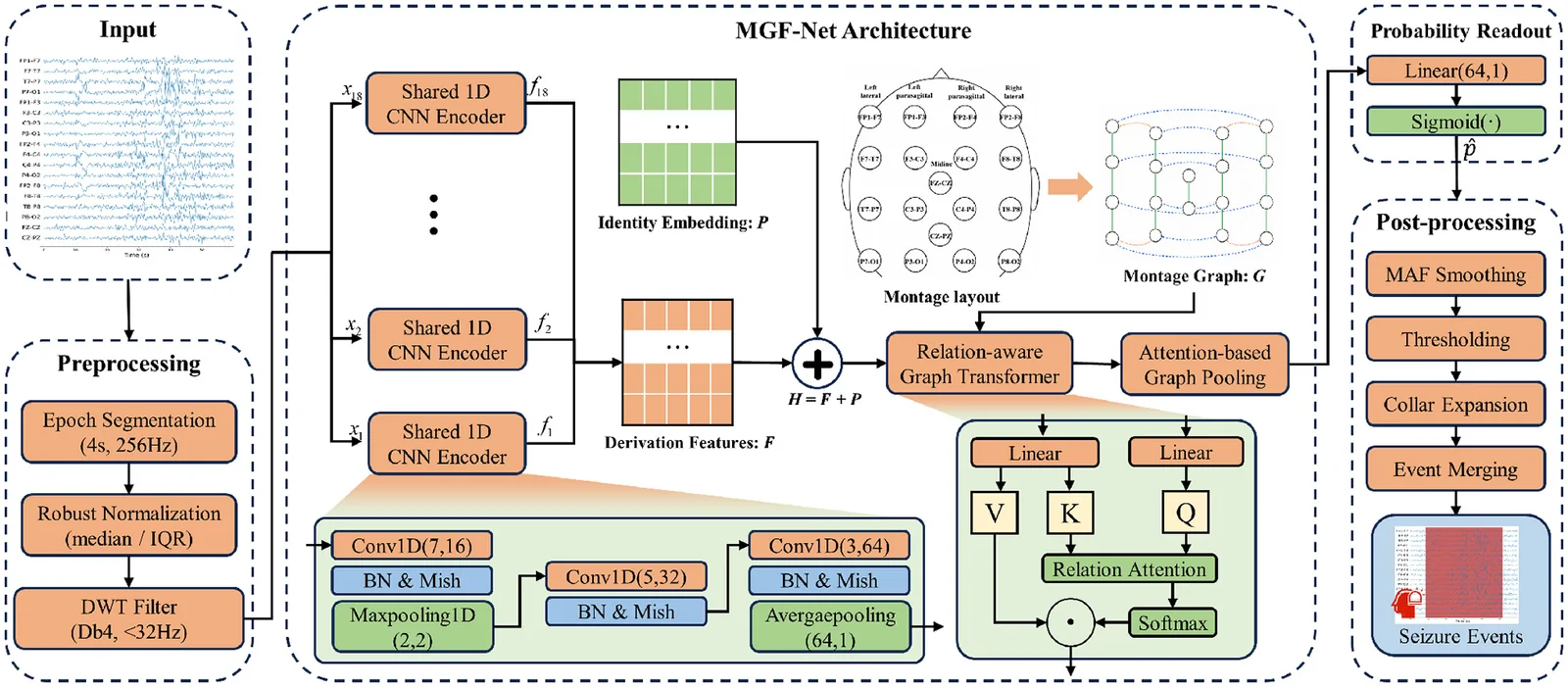
MGF-Net framework for long-term scalp EEG seizure detection.

MGF-Net is designed for seizure detection from long-term scalp EEG. The model identifies ictal patterns within individual bipolar derivations and evaluates them in the context of activity recorded from related derivations. As illustrated in Figure 2, each input epoch is processed by a shared CNN, a relation-aware graph Transformer, and graph pooling to produce an ictal probability.

### 3.1 Data preprocessing

Each EDF recording is converted to microvolts and represented using the fixed set of 18 bipolar derivations listed in Table 1. Recordings lacking one or more required derivations are excluded. The CHB-MIT recordings used in this study are sampled at 256 Hz.

**Table 1:** Bipolar derivations used as nodes of the montage graph.

Chain	Bipolar derivations
Left lateral	FP1-F7, F7-T7, T7-P7, P7-O1
Left parasagittal	FP1-F3, F3-C3, C3-P3, P3-O1
Right parasagittal	FP2-F4, F4-C4, C4-P4, P4-O2
Right lateral	FP2-F8, F8-T8, T8-P8, P8-O2
Midline	FZ-CZ, CZ-PZ

Each recording is partitioned into 4-s epochs before normalization and wavelet processing. At a sampling frequency of 256 Hz, each epoch contains *T* = 1024 samples per bipolar derivation. Let $x_{c}\in {\mathbb{R}}^{T}$ denote the signal segment from derivation *c* within one epoch, where *c* = 1, …, *C* and *C* = 18.

Robust normalization is applied independently to each derivation within each epoch, as defined in Equation 1:


$$\begin{array}{l} {\tilde{x}}_{c}=\frac{x_{c}-\mathrm{median}\left(x_{c}\right)}{\mathrm{IQR}\left(x_{c}\right)+\epsilon },\;c=1,\ldots ,C, \end{array}$$ (1)

where IQR(·) denotes the interquartile range and ϵ = 10^−6^ is used to ensure numerical stability. The normalized signals are clipped to [−5, 5] to reduce the influence of extreme amplitudes.

A five-level discrete wavelet transform is then applied independently to each clipped epoch using the Db4 wavelet and symmetric signal extension. At 256 Hz, the level-5 approximation coefficients *cA*_5_ and the level-5, level-4, and level-3 detail coefficients *cD*_5_, *cD*_4_, and *cD*_3_ correspond approximately to the 0–4, 4–8, 8–16, and 16–32 Hz bands, respectively. These coefficients are retained for signal reconstruction, whereas *cD*_2_ and *cD*_1_, which correspond approximately to the 32–64 and 64–128 Hz bands, are discarded. The reconstructed epoch therefore retains components approximately within 0–32 Hz.

The reconstructed derivation segments are stacked row-wise to form the model input *X* ∈ ℝ^*C*×*T*^. All normalization statistics and wavelet coefficients are computed independently within the epoch. Therefore, the preprocessing and network inference stages can be performed sequentially.

### 3.2 MGF-Net architecture

MGF-Net combines temporal features extracted from individual bipolar derivations with information from related derivations, as defined by the bipolar montage. The shared CNN extracts features from each derivation. The relation-aware graph Transformer uses montage-derived relation labels to guide interactions among these features. Finally, graph pooling combines the updated features from all derivations into a single feature vector for the epoch, which is used to estimate the ictal probability.

#### 3.2.1 Shared derivation encoder

The same 1D CNN is applied to every bipolar derivation to detect local temporal patterns using a single shared set of feature extractors. Sharing the encoder maps features from all derivations into a comparable representation space, whereas derivation identity is subsequently encoded by a learnable identity embedding and the montage relations. Specifically, the encoder maps $x_{c}\in {\mathbb{R}}^{T}$ to a *d*-dimensional feature vector $f_{c}=f_{\theta }\left(x_{c}\right)\in {\mathbb{R}}^{d}$, where *d* = 64 and *f*_θ_ denotes the shared CNN. The parameter set θ is shared across all derivations rather than being independently defined for each derivation. During end-to-end training, gradient contributions propagated through all derivation branches are jointly used to update this shared parameter set together with the remaining learnable components of MGF-Net. With each *f*_*c*_ treated as a column vector, the derivation features are arranged row-wise as $F=\left[f_{1}^{⊤};\ldots ;f_{C}^{⊤}\right]\in {\mathbb{R}}^{C\times d}$. The encoder contains three convolutional stages and is summarized in Table 2.

**Table 2:** Architecture of the shared 1D CNN applied to each bipolar derivation.

Layer	Output channels	Kernel/Pool	Stride	Padding	Output size
Input	1	–	–	–	1 × 1, 024
Conv1D + BN + Mish	16	7	2	3	16 × 512
MaxPooling1D	–	2	2	–	16 × 256
Conv1D + BN + Mish	32	5	2	2	32 × 128
Conv1D + BN + Mish	64	3	2	1	64 × 64
Global average pooling	–	–	–	–	64 × 1

#### 3.2.2 Montage graph construction

The independent CNN encoding does not by itself represent how bipolar derivations are related. To introduce this structural prior, the 18 derivations are treated as nodes in a complete graph, with each node pair assigned a relation label. For each node pair (*i, j*), where *i, j* = 1, …, *C*, the symmetric relation matrix *R* ∈ {0, 1, 2, 3}^*C*×*C*^ is defined as


$$\begin{array}{l} R_{ij}=\{\begin{array}{ll} 1, & i\neq j\;\text{and the derivations are adjacent in the same}\\ \; & \text{chain},\\ 2, & i\neq j\;\text{and the derivations occupy corresponding}\\ \; & \text{left–right positions},\\ 3, & i\neq j\;\text{and the derivations share exactly one electrode},\\ 0, & i=j\;\text{or otherwise}. \end{array} \end{array}$$ (2)

The conditions in Equation 2 are evaluated in the listed order. Thus, same-chain adjacency takes priority when more than one relation applies. Relation 0 is the default relation and includes self-pairs as well as node pairs without an explicitly encoded montage relation. Corresponding derivations are paired positions in the left and right lateral chains or in the left and right parasagittal chains. Because the derivations follow the same fixed order in every input, *R* is identical for all recordings.

#### 3.2.3 Relation-aware graph transformer fusion

The relation matrix specifies the type of montage relationship between two derivations, but its relevance can vary with the EEG patterns present in a particular epoch. The graph Transformer therefore combines this fixed structural prior with attention scores computed from the node features of the current epoch. A learnable derivation identity embedding *P* ∈ ℝ^*C*×*d*^ is added to *F* to obtain the initial node representation *H*^(0)^ = *F*+*P*. For each layer ℓ, let $H^{\left(\ell \right)}=\left[{\left(h_{1}^{\left(\ell \right)}\right)}^{⊤};\ldots ;{\left(h_{C}^{\left(\ell \right)}\right)}^{⊤}\right]\in {\mathbb{R}}^{C\times d}$, where $h_{i}^{\left(\ell \right)}\in {\mathbb{R}}^{d}$ is the column-vector representation of node *i*.

For attention head *a* = 1, …, *N*_h_, a query is generated using a shared projection, whereas the key and value projection matrices are selected according to the relation label *R*_*ij*_, as defined in Equation 3:


$$\begin{array}{c} \begin{array}{cc} q_{i}^{\left(a,\ell \right)} & =W_{Q}^{\left(a,\ell \right)}\mathrm{LN}\left(h_{i}^{\left(\ell \right)}\right),\\ k_{ij}^{\left(a,\ell \right)} & =W_{K,R_{ij}}^{\left(a,\ell \right)}\mathrm{LN}\left(h_{j}^{\left(\ell \right)}\right),\\ v_{ij}^{\left(a,\ell \right)} & =W_{V,R_{ij}}^{\left(a,\ell \right)}\mathrm{LN}\left(h_{j}^{\left(\ell \right)}\right). \end{array} \end{array}$$ (3)

Here, LN(·) denotes layer normalization, ℓ = 0, …, *L*_*g*_−1, and *d*_*h*_ = *d*/*N*_h_ is the feature dimension of each head. The matrices $W_{Q}^{\left(a,\ell \right)}$, $W_{K,r}^{\left(a,\ell \right)}$, and $W_{V,r}^{\left(a,\ell \right)}$ are learnable projection matrices in ${\mathbb{R}}^{d_{h}\times d}$, where *r* ∈ {0, 1, 2, 3} indexes the relation type.

The message received by node *i* from head *a* is given by Equation 4:


$$\begin{array}{c} m_{i}^{\left(a,\ell \right)}=\overset{C}{\underset{j=1}{\sum }}{\mathrm{softmax}}_{j}\left(\frac{{\left(q_{i}^{\left(a,\ell \right)}\right)}^{⊤}k_{ij}^{\left(a,\ell \right)}}{\sqrt{d_{h}}}\right)v_{ij}^{\left(a,\ell \right)}, \end{array}$$ (4)

where softmax_*j*_(·) normalizes the attention scores over all source nodes *j*. Thus, the montage relation influences both the estimated importance of node *j* and the information it transmits to node *i*.

The messages from all heads are concatenated and projected to obtain the cross-derivation update in Equation 5:


$$\begin{array}{c} u_{i}^{\left(\ell \right)}=W_{O}^{\left(\ell \right)}\mathrm{Concat}\left(m_{i}^{\left(1,\ell \right)},\ldots ,m_{i}^{\left(N_{\text{h}},\ell \right)}\right), \end{array}$$ (5)

where $W_{O}^{\left(\ell \right)}\in {\mathbb{R}}^{d\times d}$ is a learnable output projection. Because each $m_{i}^{\left(a,\ell \right)}\in {\mathbb{R}}^{d_{h}}$ and *N*_h_*d*_*h*_ = *d*, both $u_{i}^{\left(\ell \right)}$ and $h_{i}^{\left(\ell \right)}$ belong to ℝ^*d*^. The residual connection and feed-forward refinement are defined in Equations 6 and 7, respectively:


$$\begin{array}{c} {\tilde{h}}_{i}^{\left(\ell \right)}=h_{i}^{\left(\ell \right)}+u_{i}^{\left(\ell \right)}, \end{array}$$ (6)

$$\begin{array}{c} h_{i}^{\left(\ell +1\right)}={\tilde{h}}_{i}^{\left(\ell \right)}+{\mathrm{FFN}}^{\left(\ell \right)}\left({\tilde{h}}_{i}^{\left(\ell \right)}\right). \end{array}$$ (7)

Here, FFN^(ℓ)^:ℝ^*d*^ → ℝ^*d*^ denotes the feed-forward network in layer ℓ. The present model uses two graph Transformer layers (*L*_*g*_ = 2) and four attention heads (*N*_h_ = 4).

#### 3.2.4 Attention-based graph pooling and probability readout

The contribution of each derivation to a seizure decision can vary across epochs. Instead of averaging all node representations equally, the pooling module learns an importance weight for each final node representation. Let $h_{c}^{\left(L_{g}\right)}\in {\mathbb{R}}^{d}$ denote the final column-vector representation of derivation *c*. Its scalar pooling score is defined in Equation 8:


$$\begin{array}{c} s_{c}=w_{2}^{⊤}\mathrm{Mish}\left(W_{1}h_{c}^{\left(L_{g}\right)}+b_{1}\right),\;c=1,\ldots ,C, \end{array}$$ (8)

where $W_{1}\in {\mathbb{R}}^{d\times d}$, $b_{1}\in {\mathbb{R}}^{d}$, and $w_{2}\in {\mathbb{R}}^{d}$ are learnable pooling parameters. With $\text{s}={\left[s_{1},\ldots ,s_{C}\right]}^{⊤}$, the normalized importance weight of derivation *c* is α_*c*_ = softmax_*c*_(**s**). The pooled epoch representation is given by Equation 9:


$$\begin{array}{c} g=\overset{C}{\underset{c=1}{\sum }}\alpha_{c}h_{c}^{\left(L_{g}\right)}. \end{array}$$ (9)

Finally, the classifier maps g to the ictal probability, as defined in Equation 10:


$$\begin{array}{c} \hat{p}=\sigma \left(w^{⊤}g+b\right), \end{array}$$ (10)

where *w* ∈ ℝ^*d*^ and *b* ∈ ℝ are learnable classifier parameters, and σ(·) denotes the sigmoid function.

### 3.3 Post-processing

Epoch-wise probabilities can fluctuate over time and do not directly yield continuous seizure-event detections. To obtain event-level detections, the predicted probabilities are processed over each complete test recording. For a recording containing *M* test epochs, let ${\hat{p}}_{t}$ denote the predicted ictal probability for epoch *t*.

A centered moving-average filter (MAF) with odd length *L* = 2*K*+1 is applied to the probability sequence, as defined in Equation 11:


$$\begin{array}{c} {\bar{p}}_{t}=\frac{1}{\left|\Omega_{t}\right|}\underset{n\in \Omega_{t}}{\sum }{\hat{p}}_{t+n}, \end{array}$$ (11)

where Ω_*t*_ = {*n* ∈ ℤ∣−*K* ≤ *n* ≤ *K*, 1 ≤ *t*+*n* ≤ *M*} contains the valid offsets for epoch *t*.

The smoothed probability is thresholded according to Equation 12:


$$\begin{array}{c} {ŷ}_{t}=𝕀\left({\bar{p}}_{t}\geq \theta \right), \end{array}$$ (12)

where θ is the decision threshold and *I*(·) is the indicator function. Consecutive positive decisions in ŷ_*t*_ form candidate seizure events.

For a candidate event spanning epoch indices [*a, b*], a collar operation expands its boundaries according to Equation 13:


$$\begin{array}{c} \left[a^{\prime },b^{\prime }\right]=\left[\max\left(1,a-K\right),\min\left(M,b+K\right)\right], \end{array}$$ (13)

where *K* = (*L* − 1)/2 is the half-window length of the moving-average filter. The collar operation compensates for boundary attenuation caused by smoothing and thresholding. Its corresponding duration depends on the test-epoch hop duration.

**Figure 3:**
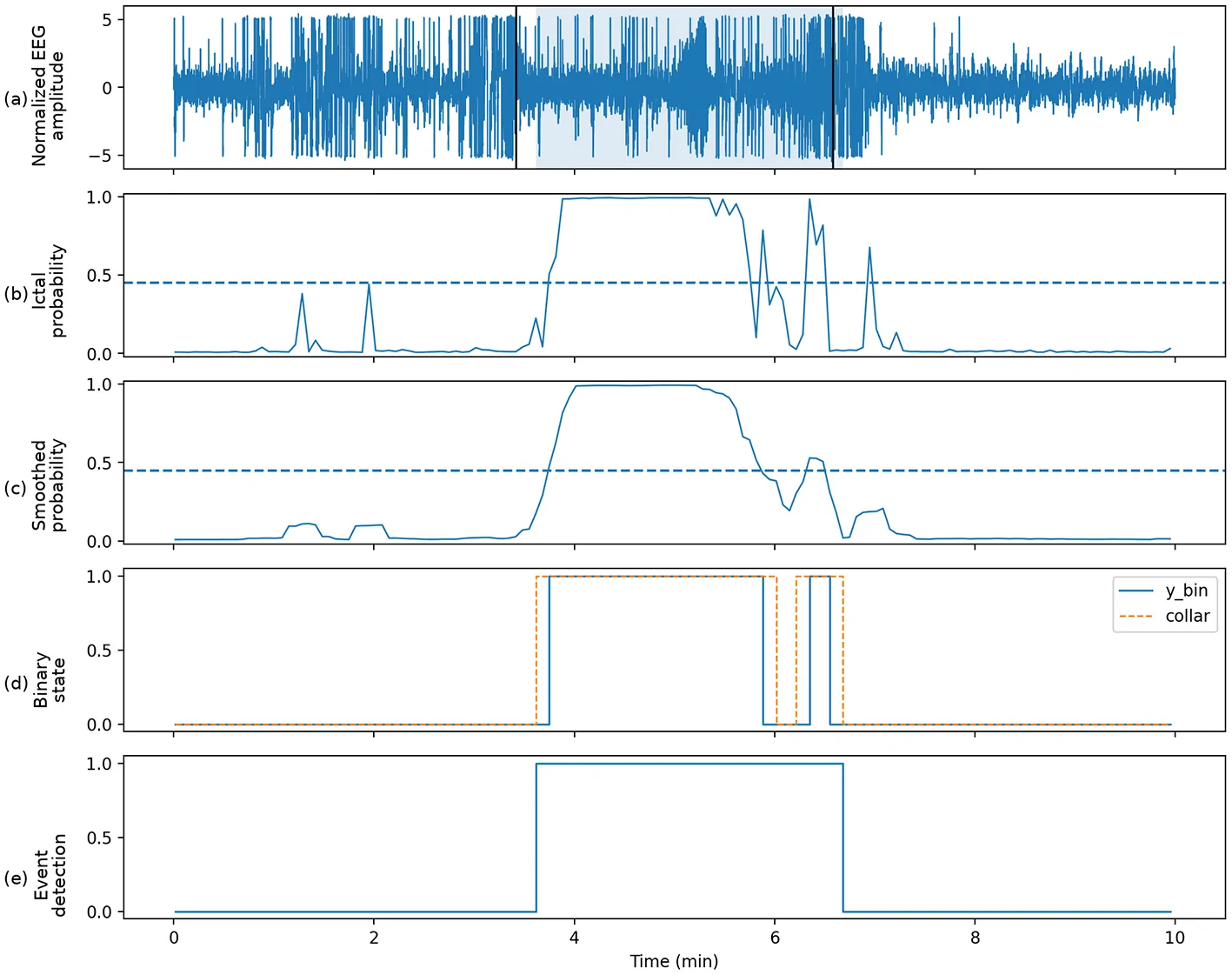
Illustration of post-processing for one representative test recording. **(a)** Example EEG trace, with the expert-annotated seizure interval indicated by black vertical lines. **(b)** Epoch-wise ictal probabilities produced by MGF-Net. **(c)** Probabilities after centered moving-average filtering, with the decision threshold θ indicated by the dashed line. **(d)** Binary decisions after thresholding and candidate events after collar expansion by *K* = (*L* − 1)/2 epochs on each side. **(e)** Final seizure-event detection after merging expanded candidates separated by no more than 60 s.

After collar expansion, two candidate events are merged when the temporal gap between their expanded intervals does not exceed 60 s. The merged intervals constitute the final seizure-event detections. Figure 3 illustrates the complete procedure.

The moving-average length *L* and decision threshold θ are selected using validation recordings only and are fixed before evaluation on the test set.

## 4 Results

### 4.1 Experimental protocol

We evaluated MGF-Net on the CHB-MIT scalp EEG dataset, which contains long-term EEG recordings in EDF format with expert-annotated seizure onset and offset times (Goldberger et al., 2000; Prasanna et al., 2021). All recordings were sampled at 256 Hz. Each retained recording was converted to microvolts and represented using the 18 fixed bipolar derivations listed in Table 1. Cases chb06, chb12, and chb16 were excluded because the seizures in chb06 and chb16 were too short for the fixed 4-s epoching protocol, whereas the changing channel configurations in chb12 prevented consistent construction of the 18 fixed bipolar derivations. The final analysis included 21 cases.

#### 4.1.1 Experimental setup

Before epoch extraction, EDF files were partitioned into training, validation, and test subsets separately for each case. This protocol used an EDF-level train/validation/test partition within each case rather than *k*-fold cross-validation or leave-one-subject-out evaluation. Seizure-containing files were randomly assigned to the training, validation, or test subsets, while retaining at least one seizure-containing file in both the training and test subsets. Seizure-free files were assigned to the test subset. The resulting case-wise partition statistics are summarized in Table 3.

**Table 3:** Case-wise statistics of the EDF-level training, validation, and test partition used in this study.

Case	Train EDFs	Validation EDFs	Test EDFs	Train seizures	Validation seizures	Test seizures	Test duration (h)
chb01	2	1	39	2	1	4	37.55
chb02	1	1	34	1	1	1	33.27
chb03	1	1	36	1	1	5	36.00
chb04	1	1	40	1	1	2	149.42
chb05	2	1	36	2	1	2	36.00
chb07	1	1	17	1	1	1	59.05
chb08	1	1	18	1	1	3	18.01
chb09	1	1	17	2	1	1	62.29
chb10	3	1	21	3	1	3	42.01
chb11	1	1	33	1	1	1	33.00
chb13	4	1	28	6	1	5	28.00
chb14	5	1	20	6	1	1	20.00
chb15	2	1	37	8	1	11	37.01
chb17	1	1	19	1	1	1	19.00
chb18	3	1	32	3	1	2	32.00
chb19	1	1	28	1	1	1	27.93
chb20	2	1	26	4	1	3	24.60
chb21	1	1	31	1	1	2	30.83
chb22	1	1	29	1	1	1	29.00
chb23	1	1	7	4	2	1	19.68
chb24	1	1	20	1	1	14	19.30
**Total**	**36**	**21**	**568**	**51**	**22**	**65**	**793.95**

Bold values indicate the totals across all included cases.

Within the training files, ictal epochs were extracted using a 4-s window with a 2-s hop, and only epochs fully contained within annotated seizure intervals were retained. Non-seizure epochs were extracted from the complementary intervals outside all annotated seizure intervals using a 4-s hop. The non-seizure epochs were then randomly subsampled to maintain a negative-to-positive epoch ratio of at most 8:1. Validation and test recordings were segmented sequentially using non-overlapping 4-s windows, and no subsampling was applied to the validation or test epochs. Because the EDF partition was completed before epoch extraction, epochs derived from the same recording could not occur in different subsets, preventing direct within-recording sample or overlapping-window leakage across the training, validation, and test data. However, different recordings from the same case could be included in different subsets; therefore, this protocol should be interpreted as recording-level rather than subject-independent evaluation.

All experiments were conducted on a workstation equipped with an NVIDIA GeForce RTX 4090 GPU with 24 GB of memory, a 13th Gen Intel Core i9-13900K CPU, and 32 GB of RAM. MGF-Net was optimized using the Adam optimizer and binary cross-entropy loss, with a learning rate of 0.01, a batch size of 256, a dropout rate of 0.2, and a maximum of 80 training epochs. The checkpoint with the lowest segment-level binary cross-entropy loss on the validation set, computed over the validation epochs before post-processing, was retained, and early stopping was applied after 15 consecutive epochs without validation-loss improvement. The derivation feature dimension was set to 64, and the relation-aware graph Transformer comprised two layers with four attention heads per layer. For post-processing, the moving-average length was selected from *L* ∈ {3, 5, 7, 9}, and the decision threshold was selected from a predefined grid over [0.3, 0.7] using the validation data. The parameter pair that maximized validation balanced accuracy was retained. The collar radius was set to *K* = (*L* − 1)/2, and collar-expanded events separated by no more than 60 s were merged. All model and post-processing parameters were fixed before test evaluation.

#### 4.1.2 Evaluation metrics

Performance was assessed at both the segment and event levels, consistent with the need for standardized evaluation of EEG-based automated seizure detection algorithms (Dan et al., 2025). Segment-level sensitivity, specificity, and accuracy were computed from the true-positive, true-negative, false-positive, and false-negative epoch counts. Because seizure and non-seizure epochs are highly imbalanced in long-term EEG, sensitivity and specificity were considered separately, and balanced accuracy was used for validation-based post-processing selection to give equal weight to the two classes. At the event level, an annotated seizure was considered detected when at least one predicted event overlapped its temporal interval, whereas a predicted event without overlap with any annotated seizure was counted as a false detection. Event sensitivity was defined as the proportion of detected seizure events, and the false detection rate (FDR) was defined as the number of false detections per hour of test EEG. For each detected seizure, latency was calculated as the difference between the annotated seizure onset and the onset of the earliest overlapping predicted event, with negative values clipped to zero. All metrics were computed separately for each case and reported as averages across cases.

### 4.2 Overall performance

Table 4 summarizes the case-wise performance of MGF-Net on the CHB-MIT dataset. Across 793.95 h of test EEG containing 65 annotated seizure events, MGF-Net achieves case-averaged segment-level sensitivity, specificity, and accuracy of 93.18%, 98.41%, and 98.38%, respectively. Segment specificity exceeds 97% in 19 of the 21 cases, demonstrating stable discrimination between seizure and non-seizure EEG segments across long-term recordings.

**Table 4:** Case-wise seizure detection performance of MGF-Net on the CHB-MIT dataset.

Case	Test (h)	TestSeiz	Det.	Segment-level			**Event-level**		
				SegSens (%)	SegSpec (%)	SegAcc (%)	Sens (%)	FDR (/h)	Latency (s)
chb01	37.55	4	4	98.82	99.58	99.58	100.00	0.37	0.00
chb02	33.27	1	1	99.18	99.15	99.15	100.00	0.30	0.00
chb03	36.00	5	5	98.11	99.25	99.25	100.00	0.44	0.00
chb04	149.42	2	2	87.96	97.00	96.99	100.00	0.80	2.50
chb05	36.00	2	2	96.69	99.00	98.99	100.00	0.86	3.50
chb07	59.05	1	1	100.00	97.10	97.10	100.00	0.93	0.00
chb08	18.01	3	3	84.66	99.25	99.11	100.00	0.39	0.00
chb09	62.29	1	1	100.00	98.45	98.45	100.00	0.71	0.00
chb10	42.01	3	3	96.15	99.80	99.79	100.00	0.07	0.33
chb11	33.00	1	1	100.00	99.38	99.38	100.00	0.55	0.00
chb13	28.00	5	5	94.34	98.15	98.13	100.00	1.50	0.60
chb14	20.00	1	1	72.73	98.10	98.08	100.00	1.40	0.00
chb15	37.01	11	11	93.71	98.40	98.35	100.00	0.76	1.09
chb17	19.00	1	1	100.00	91.70	91.71	100.00	4.21	0.00
chb18	32.00	2	2	98.35	97.70	97.70	100.00	0.56	0.00
chb19	27.93	1	1	100.00	99.02	99.02	100.00	0.61	0.00
chb20	24.60	3	3	93.33	97.95	97.94	100.00	1.18	1.33
chb21	30.83	2	2	76.92	99.55	99.53	100.00	0.32	8.50
chb22	29.00	1	1	100.00	99.68	99.68	100.00	0.17	0.00
chb23	19.68	1	1	100.00	99.65	99.65	100.00	0.25	0.00
chb24	19.30	14	14	65.89	98.75	98.35	100.00	0.93	24.71
**Total**	**793.95**	**65**	**65**	**93.18**	**98.41**	**98.38**	**100.00**	**0.82**	**2.03**

Bold values indicate the overall summary across cases. Test duration, TestSeiz, and detections are summed, while the remaining metrics are averaged across cases.

At the event level, MGF-Net detects all 65 annotated seizure events and achieves an event sensitivity of 100.00%. The case-averaged FDR is 0.82/h, and the average detection latency is 2.03 s. These results show that MGF-Net maintains high segment-level accuracy while providing reliable event-level seizure detection.

Overall, MGF-Net effectively combines temporal EEG representations with montage-guided inter-derivation interaction, achieving accurate segment-level classification and reliable seizure event detection on long-term scalp EEG recordings.

### 4.3 Sensitivity analysis of post-processing parameters

**Figure 4:**
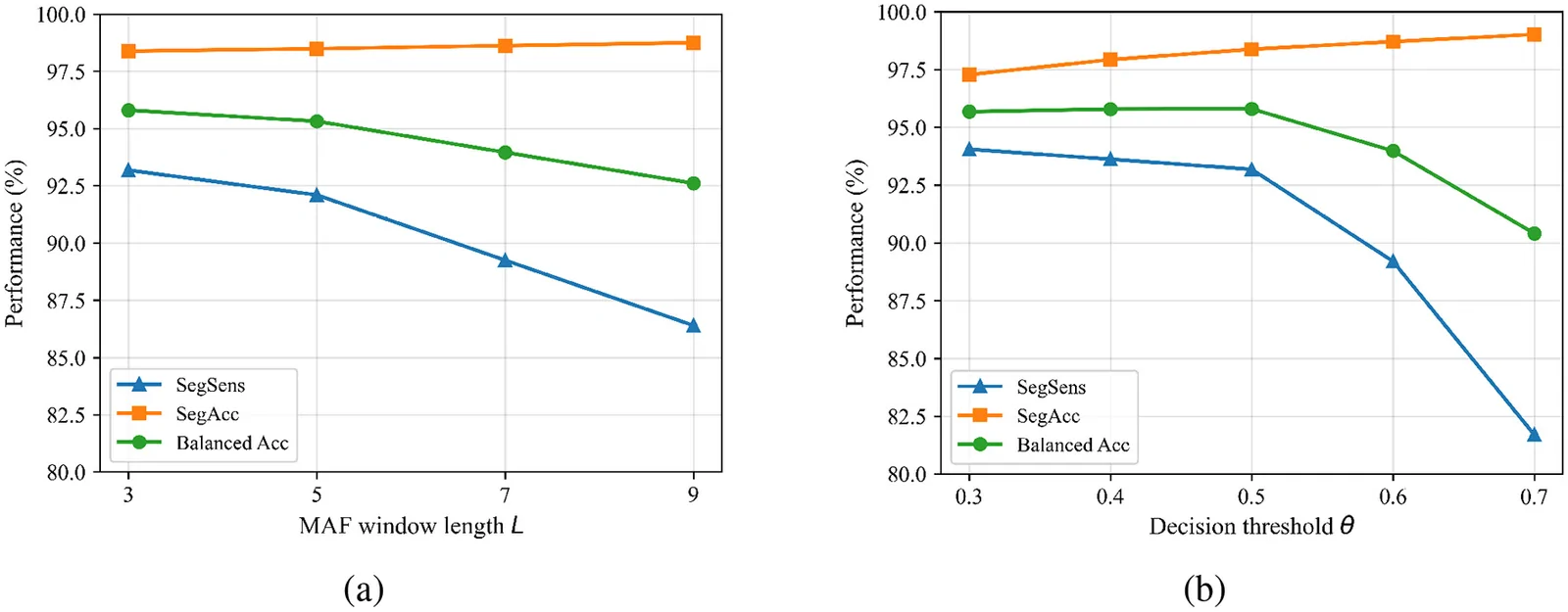
Sensitivity analysis of post-processing parameters. **(a)** Effect of the moving-average window length *L* with the decision threshold fixed at θ = 0.5. **(b)** Effect of the decision threshold θ with the moving-average window length fixed at *L* = 3.

Figure 4 shows the sensitivity of segment-level performance to the post-processing parameters. Segment sensitivity evaluates recall of seizure epochs, segment accuracy reflects overall segment-level prediction performance, and balanced accuracy provides a class-balanced evaluation under the severe imbalance between seizure and non-seizure epochs.

Figure 4a evaluates the effect of the moving-average window length *L* with the decision threshold fixed at θ = 0.5. As *L* increased from 3 to 9, segment sensitivity decreased from 93.18% to 86.40%, whereas segment accuracy increased slightly from 98.38% to 98.75%. In contrast, balanced accuracy decreased from 95.80% to 92.61%. This indicates that excessive temporal smoothing can improve the apparent overall accuracy while suppressing seizure-related responses.

Figure 4b evaluates the effect of the decision threshold θ with the moving-average window length fixed at *L* = 3. As θ increased from 0.3 to 0.7, segment sensitivity decreased from 94.05% to 81.70%, whereas segment accuracy increased from 97.28% to 99.02%. Balanced accuracy remained stable between θ = 0.3 and θ = 0.5, with values of 95.68%, 95.79%, and 95.80%, respectively, but decreased to 93.98% at θ = 0.6 and 90.41% at θ = 0.7.

These results show that segment accuracy alone is not sufficient for selecting post-processing parameters in long-term seizure detection. Larger smoothing windows and higher thresholds may increase overall accuracy, but they can substantially reduce seizure-epoch sensitivity. Therefore, the final post-processing parameters were selected by considering class-balanced performance and seizure sensitivity rather than segment accuracy alone.

### 4.4 Ablation study

Table 5 evaluates the contributions of DWT-based preprocessing, robust normalization, montage-graph modeling, relation differentiation, Graph Transformer fusion, and attention pooling. All variants were trained and evaluated using the same data split, optimization settings, and post-processing configuration, with only the component specified in each ablation setting modified.

**Table 5:** Ablation study of the principal preprocessing and architectural components of MGF-Net on the CHB-MIT dataset.

Preprocessing	Montage graph	Relation	Inter-derivation fusion	Pooling	Segment-level			Event-level		
					SegSens (%)	SegSpec (%)	SegAcc (%)	Sens (%)	FDR (/h)	Latency (s)
w/o DWT	✓	✓	Graph Transformer	Attention	92.46	98.28	98.25	99.05	0.96	2.29
w/o robust norm.	✓	✓	Graph Transformer	Attention	92.82	98.34	98.32	100.00	0.89	2.15
Full	×	—	CNN-based fusion	Attention	89.91	97.71	97.68	94.76	1.39	3.58
Full	✓	×	Graph Transformer	Attention	91.43	98.12	98.08	97.86	1.04	2.43
Full	✓	✓	R-GCN	Attention	90.84	98.03	98.00	96.43	1.19	2.74
Full	✓	✓	Graph Transformer	Mean	92.37	98.32	98.30	99.05	0.94	2.31
Full	✓	✓	Graph Transformer	Attention	**93.18**	**98.41**	**98.38**	**100.00**	**0.82**	**2.03**

Bold values indicate the results of the full MGF-Net configuration.

Removing DWT-based signal reconstruction decreases segment sensitivity, specificity, and accuracy from 93.18%, 98.41%, and 98.38% to 92.46%, 98.28%, and 98.25%, respectively. Event sensitivity decreases from 100.00% to 99.05%, while the FDR increases from 0.82/h to 0.96/h and the detection latency increases from 2.03 s to 2.29 s. Removing robust normalization produces a smaller but consistent degradation in segment-level performance, with segment sensitivity, specificity, and accuracy decreasing to 92.82%, 98.34%, and 98.32%, respectively. Although event sensitivity remains at 100.00%, the FDR increases to 0.89/h and the latency to 2.15 s. These results indicate that both preprocessing components contribute to the final detection performance, with DWT-based reconstruction showing a comparatively larger effect under the present experimental setting.

When the montage graph is removed and cross-derivation information is fused using a CNN-based module, segment sensitivity decreases from 93.18% to 89.91%, while event sensitivity decreases from 100.00% to 94.76%. The FDR and detection latency increase to 1.39/h and 3.58 s, respectively. These results support representing bipolar derivations as graph nodes rather than relying solely on convolution-based feature fusion.

When the Graph Transformer is retained, but relation differentiation is removed, segment sensitivity, specificity, and accuracy decrease to 91.43%, 98.12%, and 98.08%, respectively. Event sensitivity decreases to 97.86%, accompanied by an FDR of 1.04/h and a latency of 2.43 s. This comparison shows that explicitly distinguishing montage-derived relationships among bipolar derivations improves inter-derivation feature fusion.

Replacing the Graph Transformer with an R-GCN reduces segment sensitivity to 90.84% and event sensitivity to 96.43%, while increasing the FDR to 1.19/h and the latency to 2.74 s. Under the present montage graph, Graph Transformer fusion therefore enables more effective inter-derivation interaction than relation-aware graph convolution.

Replacing attention pooling with mean pooling also reduces performance. The mean-pooling variant achieves a segment sensitivity of 92.37%, an event sensitivity of 99.05%, an FDR of 0.94/h, and a latency of 2.31 s. This result indicates that attention pooling provides more effective aggregation of derivation-level features than uniform averaging.

Overall, the ablation results show that both preprocessing and architectural components contribute to the performance of MGF-Net. In particular, the full configuration consistently achieves the strongest overall balance across segment-level sensitivity, specificity, and accuracy, as well as event-level sensitivity, FDR, and detection latency.

### 4.5 Comparison with representative methods

Table 6 compares MGF-Net with representative seizure detection methods on the CHB-MIT dataset. To ensure a controlled and fair comparison, all baseline methods were reimplemented and evaluated using the same EDF-file training/validation/test partition, post-processing protocol, and evaluation metrics as MGF-Net. Thus, the results reported in Table 6 were obtained under a unified experimental protocol rather than being directly taken from the originally reported values. The listed methods include recurrent neural networks, convolutional–recurrent neural networks, temporal convolutional networks, Transformer-based detectors, and the graph-based TGCN method. TGCN represents a graph-based strategy that incorporates predefined EEG lead topology. In contrast, CNN-Informer and MCLF represent two distinct strategies for multichannel EEG modeling: CNN-Informer applies multichannel convolutional encoding followed by Informer-based temporal modeling, whereas MCLF integrates multichannel information through channel serialization and liquid-state evolution.

**Table 6:** Comparison of MGF-Net with representative seizure detection methods on the CHB-MIT dataset.

References	Method	Segment-level			Event-level		
		SegAcc (%)	SegSens (%)	SegSpec (%)	Sens (%)	FDR (/h)	Latency (s)
Covert et al. (2019)	TGCN	96.95	87.31	97.01	93.76	1.43	3.57
Zhang et al. (2022)	Bi-GRU	96.18	83.40	96.22	90.41	1.71	2.93
Zhou et al. (2023)	STFT + CNN-LSTM	96.11	82.15	96.19	90.52	2.51	6.78
Zhu and Wang (2023)	CNN-Transformer	96.61	86.03	96.65	92.22	1.34	3.66
Dong et al. (2024)	TCN-BiLSTM	96.63	85.99	96.69	92.18	1.37	4.46
(Li C. et al. 2025)	CNN-Informer	97.17	88.02	97.23	94.87	1.17	3.31
Wang et al. (2026)	MCLF	98.18	91.53	98.22	100.00	0.98	2.33
Our work	MGF-Net	**98.38**	**93.18**	**98.41**	**100.00**	**0.82**	**2.03**

Bold values indicate the results of the proposed MGF-Net.

#### 4.5.1 Segment-level results

MGF-Net achieves a segment-level accuracy of 98.38%, a sensitivity of 93.18%, and a specificity of 98.41%. Compared with MCLF, MGF-Net improves accuracy from 98.18% to 98.38%, sensitivity from 91.53% to 93.18%, and specificity from 98.22% to 98.41%. These improvements indicate that explicitly modeling montage-derived relationships provides a more informative fusion mechanism than organizing derivation features only as a serialized sequence.

Among the remaining methods, CNN-Informer achieved the strongest segment-level performance, with an accuracy of 97.17%, a sensitivity of 88.02%, and a specificity of 97.23%. Compared with CNN-Informer, MGF-Net improves accuracy by 1.21 percentage points, sensitivity by 5.16 percentage points, and specificity by 1.18 percentage points. This comparison suggests that explicit relation-aware interaction among bipolar derivations can complement joint multichannel convolutional encoding and temporal feature learning. Compared with the graph-based TGCN, which achieved an accuracy of 96.95%, a sensitivity of 87.31%, and a specificity of 97.01%, MGF-Net improves the corresponding metrics by 1.43, 5.87, and 1.40 percentage points, respectively. This result further supports the benefit of explicitly distinguishing montage-derived relation types during cross-derivation graph modeling.

#### 4.5.2 Event-level results

MGF-Net detects all annotated seizure events in the test data, achieving an event sensitivity of 100.00%, an average FDR of 0.82/h, and an average latency of 2.03 s. Compared with MCLF, MGF-Net maintains the same event sensitivity while reducing the FDR from 0.98/h to 0.82/h and shortening the latency from 2.33 s to 2.03 s.

Relative to the CNN-Informer results, MGF-Net improves event sensitivity from 94.87% to 100.00%, reduces the FDR from 1.17/h to 0.82/h, and shortens the latency from 3.31 s to 2.03 s. Relative to TGCN, MGF-Net increases event sensitivity from 93.76% to 100.00%, reduces the FDR from 1.43/h to 0.82/h, and shortens the latency from 3.57 s to 2.03 s. Taken together, the comparisons with MCLF and CNN-Informer suggest that explicitly preserving derivation-specific representations and modeling their montage-defined interactions provides advantages over both serialized multichannel modeling and joint convolutional multichannel encoding.

Overall, the comparative results show that montage-guided graph modeling provides an effective complement to temporal EEG feature extraction. Compared with predefined-topology graph modeling, joint multichannel convolutional encoding, and channel-serialization-based fusion, MGF-Net preserves derivation-specific temporal representations and explicitly models their montage-defined interactions. By combining shared temporal encoding, relation-aware derivation interaction, and attention-based graph pooling, MGF-Net achieves improved segment-level discrimination and event-level seizure detection on long-term scalp EEG.

### 4.6 Computational efficiency

To further assess the computational requirements of MGF-Net, we measured its model size and inference speed on the NVIDIA GeForce RTX 4090 GPU described in Section 4.1.1. Based on the architecture described in Section 3.2, MGF-Net contains 105,089 trainable parameters. With a batch size of 1, the average forward-pass inference time for one 4-s EEG epoch was 1.80 ± 0.22 ms, corresponding to approximately 555.54 epochs/s. Under batched inference with a batch size of 256, the throughput increased to approximately 56,369 epochs/s, with a peak allocated GPU memory of 378.34 MB. These measurements refer to neural-network forward inference and exclude EDF loading, preprocessing, data transfer, and post-processing. The results indicate that MGF-Net imposes a modest computational burden for long-term EEG analysis.

### 4.7 Attention-based model interpretation

**Figure 5:**
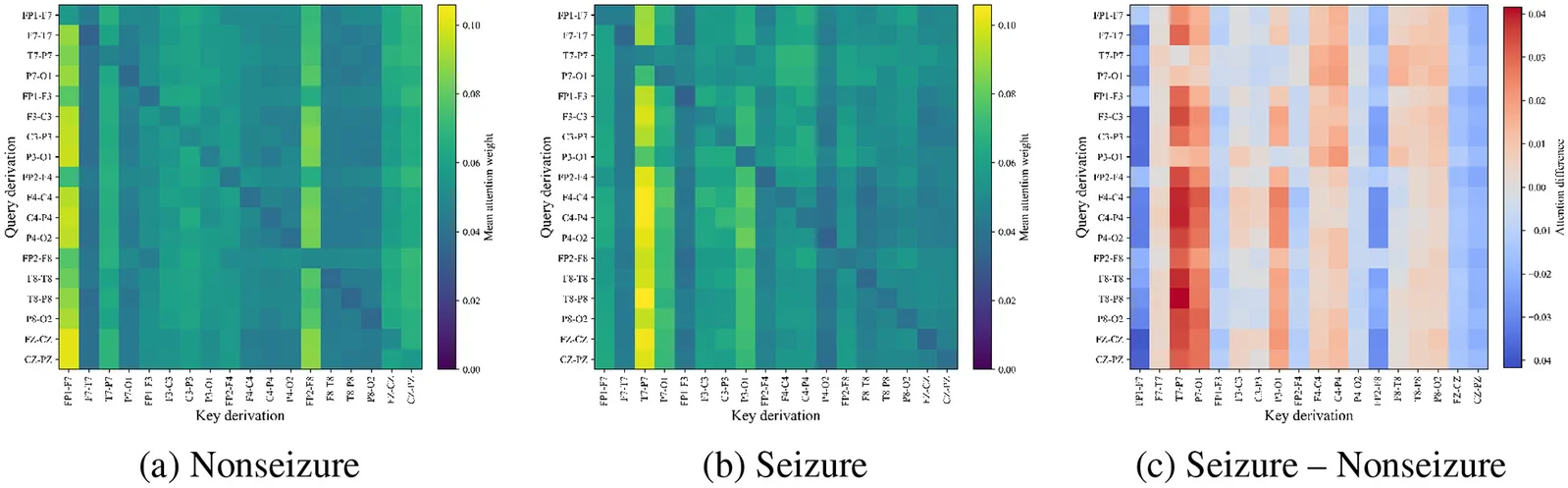
Visualization of Graph Transformer attention patterns for non-seizure and seizure epochs. **(a)** Mean cross-derivation attention matrix for non-seizure epochs. **(b)** Mean cross-derivation attention matrix for seizure epochs. **(c)** Difference between the seizure and non-seizure attention matrices. Rows correspond to query derivations and columns to key derivations. Positive values in panel **(c)** indicate relatively increased attention during seizure epochs, whereas negative values indicate decreased attention.

To further investigate how MGF-Net modulates cross-derivation interactions under different EEG states, the attention weights from the final Graph Transformer layer were extracted for seizure and non-seizure epochs in the test set. Inspection of individual attention maps showed noticeable variability across epochs; therefore, an aggregate analysis was adopted to reduce the influence of individual-example fluctuations and provide a more stable comparison between the two EEG states. For each epoch, the attention matrices were averaged across the four attention heads and then averaged separately over all seizure epochs and all non-seizure epochs in the test set. Figure 5 shows the resulting mean attention matrices for non-seizure and seizure epochs, together with their difference (seizure minus non-seizure). Each row represents a query derivation, and each column represents a key derivation; therefore, a larger value indicates that the corresponding key derivation receives greater relative attention during cross-derivation information aggregation.

To summarize the attention received by individual derivations, the mean attention for each key derivation was further obtained by averaging the corresponding column across all query derivations. The results reveal a clear redistribution of attention between non-seizure and seizure epochs. T7-P7 exhibits the largest increase in mean received attention, from 0.0641 during non-seizure epochs to 0.0934 during seizure epochs. P7-O1 also increases from 0.0448 to 0.0667, while more moderate increases are observed for P3-O1 and C4-P4. In contrast, the mean attention received by FP1-F7 decreases from 0.0883 to 0.0602, while FP2-F8 and CZ-PZ also receive relatively lower attention during seizure epochs. These changes are further highlighted in the difference map.

Importantly, the observed pattern reflects a redistribution rather than a uniform increase in attention, because the attention weights for each query derivation are normalized by the softmax operation. Thus, although the montage-derived relation matrix is fixed across recordings, the effective cross-derivation attention pattern remains data-dependent and varies with the EEG state. This analysis provides additional insight into the adaptive information fusion performed by the relation-aware Graph Transformer. The observed attention patterns are interpreted as indicators of model behavior rather than direct evidence of anatomical seizure onset or clinical localization.

## 5 Discussion

The main finding of this study is that bipolar montage structure can be used as an explicit prior for multi-derivation EEG seizure detection. MGF-Net achieved strong segment-level discrimination and detected all 65 annotated seizure events across 793.95 h of test EEG, with a case-averaged FDR of 0.82/h and an average latency of 2.03 s. These results suggest that incorporating montage-defined relationships into derivation fusion can improve both epoch-level classification and recording-level seizure detection.

A central feature of MGF-Net is the representation of bipolar derivations as nodes in a relation-labeled montage graph. Rather than treating derivations only as independent channels or as elements of a serialized input sequence, the proposed graph distinguishes same-chain adjacency, left–right correspondence, and shared-electrode relations. These relations do not represent anatomical or functional connectivity; instead, they provide a fixed structural prior describing how bipolar derivations are organized within the montage. The relation-aware graph Transformer uses this prior through relation-specific key and value projections, allowing the relevance and transmitted information of each derivation to vary across epochs. The ablation results support this design: removing montage-graph modeling, removing relation differentiation, or replacing the Graph Transformer with an R-GCN consistently reduced both segment-level and event-level performance. Together, these findings indicate that explicit montage relation modeling and adaptive derivation interaction are both important for the proposed framework.

The montage-derived relation matrix is fixed across recordings; however, the cross-derivation interactions are not fully static. In the current implementation, all derivations form a complete graph, while the fixed matrix R assigns a montage-derived relation label to each node pair. The attention weights are then computed from the node features of each EEG epoch, allowing the strength of information exchange between derivations to vary dynamically with the current EEG state. Thus, MGF-Net combines a fixed and interpretable relation prior with data-dependent cross-derivation interaction.

The direct comparison with MCLF further supports the value of montage-guided fusion. Under the same experimental setting, MGF-Net improved segment-level accuracy, sensitivity, and specificity, while maintaining the same event sensitivity and reducing both FDR and detection latency. MCLF integrates multichannel information through channel serialization and liquid-state evolution, whereas MGF-Net explicitly models structural relationships among bipolar derivations before graph-level aggregation. The comparisons with the remaining baseline methods further show that MGF-Net achieves superior segment-level and event-level performance under the same experimental protocol.

Several directions remain for future work. Although the montage-derived relations provide a fixed and interpretable structural prior, the predefined relation categories do not explicitly represent patient-specific or epoch-dependent functional coupling as separate relation types. The montage prior could therefore be augmented with feature-dependent edge gating or learned relation assignments, allowing additional data-driven dependencies to be modeled while retaining the interpretability of the montage-derived structure. Because MGF-Net was evaluated only on the pediatric CHB-MIT dataset, its generalizability to adult populations, different seizure phenotypes, and recordings acquired at other institutions remain to be established. In real-world deployment, performance may also be affected by inter-center differences in recording hardware, referencing schemes, artifact characteristics, sampling configurations, and clinical montage settings. In addition, the current implementation assumes a fixed set of 18 bipolar derivations and excludes recordings with missing required derivations. Accordingly, the current model cannot be directly applied to an arbitrary four-derivation configuration without adaptation. A reduced-channel extension could construct the montage graph from the available derivations or use node masking to exclude missing derivations during graph fusion and pooling. These factors may introduce domain shifts that are not represented in the present evaluation. Further studies should therefore evaluate MGF-Net on independent multicenter cohorts and extend the framework to accommodate incomplete or alternative montages, with model adaptation or decision-threshold recalibration where necessary across recording environments.

## 6 Conclusion

This study proposed MGF-Net, a montage-guided fusion network for seizure detection from long-term scalp EEG. The proposed method represents 18 bipolar derivations as nodes in a relation-labeled graph and incorporates same-chain adjacency, left–right correspondence, and shared-electrode relations into relation-aware graph Transformer fusion. Shared one-dimensional CNN encoding extracts temporal features from individual derivations, while attention-based graph pooling generates an epoch representation for ictal probability estimation. A post-processing procedure subsequently converts epoch-wise probabilities into seizure-event detections.

Experiments on the CHB-MIT dataset showed that MGF-Net achieved a case-averaged segment-level accuracy of 98.38%, sensitivity of 93.18%, and specificity of 98.41%. The model detected all 65 annotated seizure events, yielding an event sensitivity of 100.00%, an FDR of 0.82/h, and an average latency of 2.03 s. Ablation experiments further demonstrated the contributions of montage-graph modeling, relation differentiation, graph Transformer fusion, and attention-based pooling. These findings support the use of bipolar montage structure as an explicit prior for multi-derivation EEG seizure detection.

## Data Availability

The original contributions presented in the study are included in the article/supplementary material, further inquiries can be directed to the corresponding author.
